# Imaging Nanomedicine-Based Drug Delivery: a Review of Clinical Studies

**DOI:** 10.1007/s11307-018-1255-2

**Published:** 2018-08-06

**Authors:** Francis Man, Twan Lammers, Rafael T. M. de Rosales

**Affiliations:** 1 School of Biomedical Engineering & Imaging Sciences, King’s College London, St Thomas’ Hospital, Westminster Bridge Road, London, SE1 7EH UK; 2 Department of Nanomedicine and Theranostics, Institute for Experimental Molecular Imaging (ExMI), University Clinic and Helmholtz Institute for Biomedical Engineering, RWTH Aachen University, Pauwelsstrasse 30, 52074 Aachen, Germany; 3 Department of Targeted Therapeutics, MIRA Institute for Biomedical Technology and Technical Medicine, University of Twente, P.O. Box 217, 7500 AE Enschede, The Netherlands; 4 Department of Pharmaceutics, Utrecht Institute for Pharmaceutical Sciences (UIPS), Utrecht University, Universiteitsweg 99, 3584 CG Utrecht, The Netherlands

**Keywords:** Nanomedicine, Drug delivery, Liposome, PET, SPECT, MRI, Ultrasound, Nanoparticle, Companion diagnostic, EPR

## Abstract

Imaging plays a key role in the preclinical evaluation of nanomedicine-based drug delivery systems and it has provided important insights into their mechanism of action and therapeutic effect. Its role in supporting the clinical development of nanomedicine products, however, has been less explored. In this review, we summarize clinical studies in which imaging has provided valuable information on the pharmacokinetics, biodistribution, and target site accumulation of nanomedicine-based drug delivery systems. Importantly, these studies provide convincing evidence on the uptake of nanomedicines in tumors, confirming that the enhanced permeability and retention (EPR) effect is a real phenomenon in patients, albeit with fairly high levels of inter- and intraindividual variability. It is gradually becoming clear that imaging is critically important to help address this high heterogeneity. In support of this notion, a decent correlation between nanomedicine uptake in tumors and antitumor efficacy has recently been obtained in two independent studies in patients, exemplifying that image-guided drug delivery can help to pave the way towards individualized and improved nanomedicine therapies.

## Introduction

Drug delivery systems based on nanoparticle technologies have been explored for more than 40 years, being one of the most active multidisciplinary fields of research to date [1]. Of the several drug delivery platforms available, liposomes, polymers, and solid inorganic nanoparticles have been the most widely studied. The rationale behind these drug carrier systems is to exploit their specific pharmacokinetic and biodistribution properties to deliver sufficient therapeutic amounts of the drug cargo to the specific target(s) (the drug being usually a toxic and/or insoluble small molecule), and to reduce its side effects due to local controlled release. To date, several nanomedicine drug delivery systems based on these concepts have been translated into clinical products, these include Abraxane®, DaunoXome®, Doxil®/Caelyx®, Marqibo®, Myocet®, and Onivyde® among others, with more in clinical trials (> 45 found in ClinicalTrials.gov).

Imaging has played a very important role in the progress of this field. From a developmental perspective, it allows the non-invasive measurement of the biodistribution and pharmacokinetics of these drug delivery systems in animal models of disease, allowing us to select the best candidates by providing answers to important questions such as “Where do they go inside the body?,” “How long do they stay?,” “How are they cleared?,” “Are they reaching the target? and if so, how much?,” and “Is the drug being released?.” Several imaging techniques are available to obtain such information. To gain a deeper understanding of the different preclinical imaging modalities available—with their intrinsic advantages and disadvantages—and how they have supported the preclinical development of drug delivery systems—we refer the reader to several recent reviews on this area [2–7].

In the clinical setting, imaging can play an additional role due to human and disease heterogeneity. It is widely accepted that human/disease heterogeneity affects the efficacy of all therapies and is particularly detrimental to the clinical effectiveness and translation of therapeutic nanomedicines [8–10]. Unlike in animal models of disease, where most frequently the same genetic strains of mice and disease cell lines are used to assess the efficacy of drug delivery systems, in humans, heterogeneity is present between patients with different diseases, those with the same disease, and even within different lesions of the same patient. This heterogeneity has led to nanomedicines being approved based on their improved safety profile compared to conventional drugs, rather than improvements in therapeutic efficacy [11]. Hence, to overcome this problem, imaging methods that allow us to predict at the patient-to-patient level the efficacy of drug delivery systems, as well other therapeutics, could play important roles in the future [9].

The objective of this review is to analyze the progress and future prospects in the area of imaging drug delivery in humans. Our aim is twofold: first, we want to provide a descriptive analysis to date on how different clinical imaging techniques have played an important role in providing proof-of-concept data to support the development of drug delivery systems into clinical products. Second, we aim at highlighting how recent studies are shining new light into the inter- and intra-patient heterogeneity problem and how imaging can be used to predict drug delivery/therapeutic efficacy. Where possible, we have also tried to highlight findings from all of these studies that, in our opinion, deserve further attention.

To identify clinical studies in the field of image-guided drug delivery, we searched PubMed in January 2018, using combinations of the following terms: drug delivery, imaging, liposome, MRI, nanomedicine, nanoparticle, PET, radiolabeled, scintigraphy, SPECT, and ultrasound. Results were then restricted to clinical trials and articles were manually screened for relevance (Table 1). Studies in which imaging, most frequently x-ray computed tomography (CT) and 2-deoxy-2-[^18^F]fluoro-d-glucose (FDG) positron emission tomography (PET), was used solely for monitoring response to treatment were not included. With the focus being on nanomedicine drug delivery systems, studies of labeled small molecules and antibodies were not considered. For a brief overview of antibody-based theranostics, we refer the reader to the excellent review by Moek *et al.* [32]. The results of our search showed that liposome technologies are the most studied drug delivery system in humans. For reviews on image-guided drug delivery focused more specifically on clinical applications of liposomes in combination with imaging, we refer the reader to recently published reviews by Petersen *et al.* [33] and Lamichhane *et al.* [34]. Our search results also highlighted that the majority of the clinical studies of imaging-guided drug delivery to date have been performed using nuclear imaging modalities, principally planar gamma scintigraphy with only a minority using single-photon emission computed tomography (SPECT) or PET. This was followed by fewer studies using ultrasound (US) and magnetic resonance imaging (MRI). A brief description of each imaging technique is provided in each section, and the reader will find an excellent overview and comparison of the various imaging techniques in the introduction to molecular imaging by James and Gambhir [35]. All results have been organized in two levels, imaging technique and disease.

**Table 1:** Selected clinical studies of image-guided approaches to nanomedicine drug delivery

Reference	Imaging modality	Tracer	Nanomedicine type	Drug	Disease	No. of patients	Main outcome (imaging)
Lopez-Berestein *et al.* [12]	Scintigraphy	^99m^Tc	Liposome	None	Cancer	7	Safety of ^99m^Tc-labeled liposomes
Turner *et al.* [13], Presant *et al.* [14]	Scintigraphy	^111^In	Liposome	None	Cancer	24	Liposomes for tumor detection; EPR heterogeneity
Presant *et al.* [15, 16]	Scintigraphy	^111^In	Liposome	None	Cancer	130	Liposomes for tumor detection; EPR heterogeneity
Khalifa *et al.* [17]	SPECT	^111^In	Liposome	None	Cancer	8	Tumor delineation with radiolabeled liposomes
Stewart *et al.* [18], Harrington *et al.* [19]	SPECT	^111^In	Liposome (PEG)	None	Cancer	17	Stealth liposome biodistribution; EPR heterogeneity
Koukourakis *et al.* [20]	Scintigraphy + SPECT	^99m^Tc	Liposome (PEG)	Doxorubicin	Cancer	18	Tumor uptake; EPR heterogeneity
Koukourakis *et al.* [21]	Scintigraphy + SPECT	^99m^Tc	Liposome (PEG)	Doxorubicin	Cancer	7	Tumor uptake; EPR heterogeneity
Murray *et al.* [22]	Scintigraphy	^99m^Tc	Liposome	Muramyl tripeptide phosphatidylethanolamine	Cancer	4	Tumor uptake
Giovinazzo *et al.* [23]	SPECT	^99m^Tc sulfur colloid	Liposome	Doxorubicin	Cancer	10	Feasibility of companion diagnostic approach
Dams *et al.* [24]	Scintigraphy + SPECT	^99m^Tc	Liposome (PEG)	None	Infection, inflammation	35	Sensitive method for detection of infections
Weers *et al.* [25]	Scintigraphy	^99m^Tc	Liposome	Amikacin	Infection	(healthy) 3	Use of radiolabeled liposomes for respiratory diseases
Farr *et al.* [26]	Scintigraphy	^99m^Tc	Liposome	None	Respiratory diseases	(healthy) 4	Use of radiolabeled liposomes for respiratory diseases
Bhavna *et al.* [27]	Scintigraphy	^99m^Tc	Nanoparticle	Salbutamol	Respiratory diseases	10	Lung accumulation of nanoparticulate drug
Lee *et al.* [28]	PET	^64^Cu	Liposome	Doxorubicin	Cancer	19	EPR heterogeneity; superiority of imaging *vs.* blood sampling
Phillips *et al.* [29]	PET, fluorescence	^124^I	Nanoparticle (Cornell dot)	None	Cancer	5	Rapid tumor uptake; multimodal approach useful in surgery
Ramanathan *et al.* [30]	MRI	Iron oxide nanoparticles (Ferumoxytol®)	Liposome	Irinotecan	Cancer	13	EPR heterogeneity; companion diagnostic approach
Lyon *et al.* [31]	Ultrasound		Liposome	Doxorubicin	Cancer	(planned) 28	

## Clinical Studies Using Gamma Scintigraphy and Single-Photon Emission Computed Tomography Imaging

Gamma scintigraphy and single-photon emission computed tomography (SPECT) imaging rely on gamma-emitting radioisotopes, most commonly technetium-99m (^99m^Tc, *t*_1/2_ = 6.0 h, *γ* = 140 keV), indium-111 (^111^In, *t*_1/2_ = 2.8 day, *γ* = 171 keV, 245 keV), iodine-123 (^123^I, *t*_1/2_ = 13.2 h, *γ* = 159 keV), or iodine-131 (^131^I, *t*_1/2_ = 8.0 day, *γ* = 364 keV) in the clinic. The signal is captured by a gamma camera, equipped with collimators to localize the origin of the signal. Conventional scintigraphy provides two-dimensional (2D) images. In SPECT imaging, the gamma camera is rotated around the patient to obtain multiple 2D projections which can then be reconstructed into a three-dimensional (3D) image. Current clinical SPECT scanners provide a spatial resolution of 8–10 mm, a temporal resolution of a few minutes and a sensitivity of 10^−10^ to 10^−11^ mol/l of radiotracer [35].

### Oncology

Most of the studies involved imaging liposomes radiolabeled with Tc-99m or In-111 to evaluate drug delivery to tumor sites. Following preclinical studies showing accumulation of liposomes in tumors, early clinical studies were aimed at establishing both the safety of the liposomes and their use as imaging agents for tumor detection and staging [36–39]. For example, Lopez-Berestein *et al.* [12] administered Tc-99m-labeled liposomes to seven cancer patients. It was unclear at the time whether early-generation liposomes selectively accumulated at tumor sites, and therefore liposomes were primarily seen as a means to reduce the toxicity of their payload or to target macrophages. The study revealed the accumulation of liposomes in macrophage-rich tissues such as the lungs, liver, and spleen, but did not mention any accumulation at the tumor sites. It is, however, unlikely that this could have been observed, considering that the four out of seven patients had various forms of leukemia, and one of those with a solid tumor was in complete remission. The value of this study lies more in its demonstration of the safety and relative ease of using radiolabeled liposomes in humans. The safety of the technique was confirmed in the first clinical study using In-111-labeled liposomes, conducted by Turner *et al.* [13, 14]. Although the study was unblinded, accumulation of liposomes was observed at known tumor sites in 22 out of 24 patients, and revealed unsuspected tumors in 3 patients, demonstrating the utility of radiolabeled liposomes for tumor detection. Furthermore, a high variability of liposomal uptake in tumors was noted, possibly the first observation of enhanced permeability and retention (EPR) effect heterogeneity in humans. The same group later published what remains by far the largest clinical trial of radiolabeled nanomedicines, in terms of number of patients imaged [15]. Even on this larger scale, In-111-labeled liposomes were safe to use, and the authors reported 70–80 % sensitivity and 90 % specificity for non-cerebral tumor detection. Scintigraphic images of patients with Kaposi’s sarcoma and head and neck cancer (HNC) showed accumulation of radioactivity at the tumor sites [16]. The authors concluded to the usefulness of In-111-labeled liposomes as a diagnostic tool and of liposomes in general as drug delivery vehicles. Later, Khalifa *et al.* reported low uptake but excellent tumor delineation in seven out of eight patients with high-grade glioma using In-111-labeled liposomes [17].

**Fig. 1.**
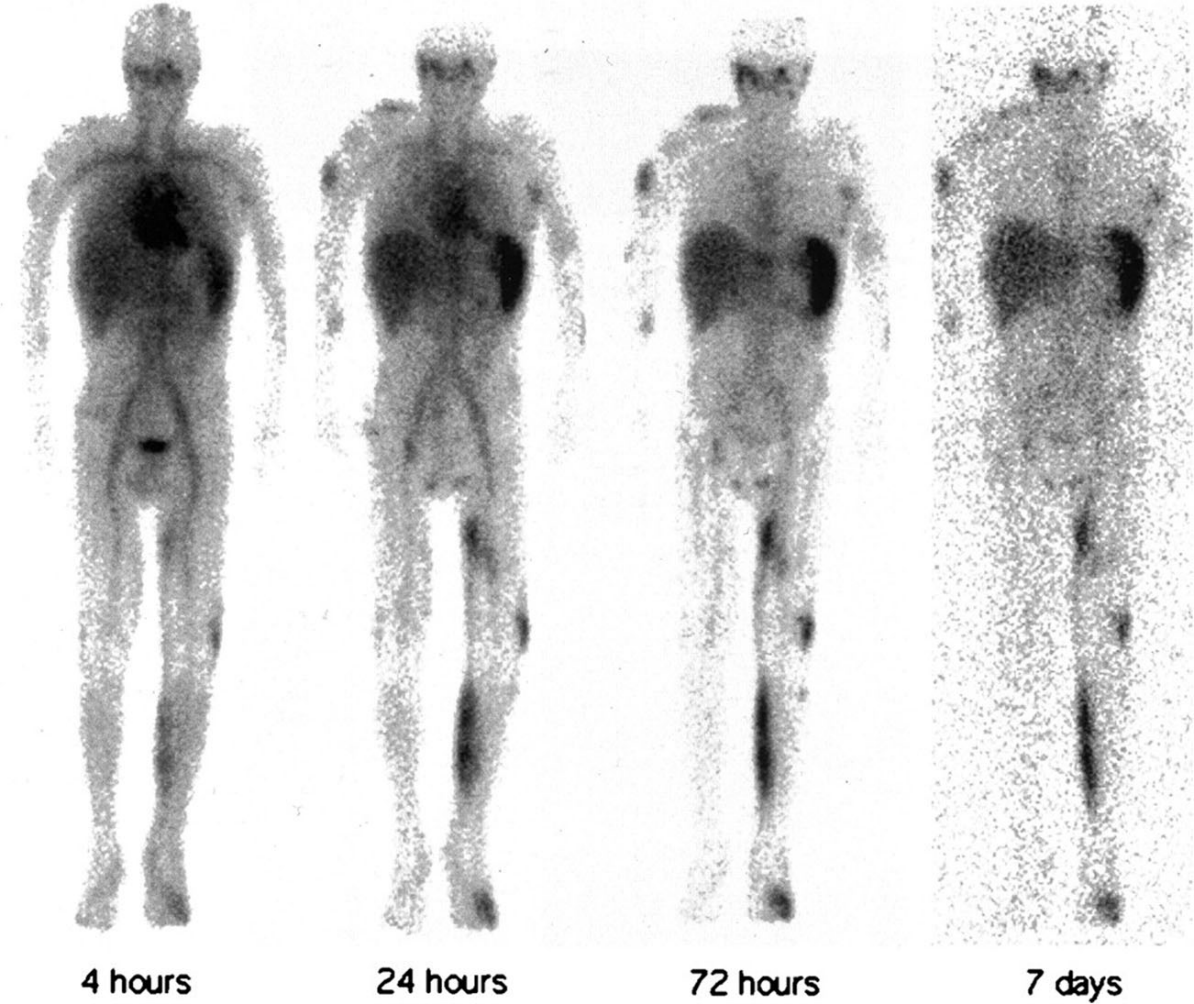
Whole-body gamma camera images over 7 days of a patient with Kaposi’s sarcoma administered In-111-labeled liposomes. Areas of liposome uptake in the left foot and leg, right arm, and face corresponded with typical Kaposi’s sarcoma lesions. Prolonged retention of the radiolabel is seen despite significant clearance of circulating liposomes, as demonstrated by disappearance of the cardiac blood pool image. Adapted with permission from the American Association for Cancer Research: Harrington *et al.* [19].

Two particularly noteworthy studies are those performed by Stewart, Harrington, and colleagues [18, 19]. By then, liposome technology had witnessed the development and clinical approval of so-called stealth, *i.e.*, polyethylene glycol (PEG)-coated, or PEGylated, liposomes with increased circulation times [40]. The first study aimed at establishing the biodistribution pattern of stealth liposomes in 17 patients with solid tumors. The extended circulation time compared to earlier-generation liposomes [41] was confirmed by scintigraphic imaging. Tumor accumulation of the radiolabeled liposomes was evident, in some cases for up to 10 days after administration, with eightfold variations in uptake between tumors (Table 2). In the second, more detailed study, 15 patients with locally advanced breast cancer, lung cancer, cervix cancer, or squamous cell HNC, or high-grade glioma, were administered In-111-labeled PEGylated liposomes, with the aim of obtaining precise information on the pharmacokinetics of these liposomes. One of the observations made was that of the daily urinary excretion of a small percentage of the injected In-111, presumably caused by the slow degradation of the liposomes in the tissues. This preliminary observation deserves further investigation. Indeed, beyond measuring liposomal uptake in the tumor, radiolabeling liposomes could provide a means of quantifying the amount of drug released from the liposomes. In the present case, In-111 urinary excretion might be used as a surrogate marker of drug release from the liposomal formulation. Scintigraphy showed a long circulation time of the liposomes and accumulation mostly in the liver and spleen. This was accompanied by a high stability of the liposomes that remained in circulation up to 4 days post-administration. Because this slow clearance from the blood led to high background signal, accumulation of the In-111-labeled liposomes in the tumors was not observed in the first 48 h; however, the tumors were eventually visualized in 15 of the 17 patients studied. One of the main findings from this trial was that liposomal accumulation in the tumors could be seen for up to a week after administration. This was demonstrated in the striking scintigraphic image of a patient with Kaposi’s sarcoma, illustrating the EPR effect in humans (Fig. 1).

**Table 2.** EPR heterogeneity: variability of radiolabeled liposome uptake in tumors. Adapted with permission from the American Association for Cancer Research: Harrington *et al.* [19]

Patient	Tumor	Stage	Whole body scan	SPECT	Total % injected dose^a^	% ID/kg^b^
1	SCC bronchus	T4N0M0	Positive	Positive	1.7	12.5
2	SCC bronchus	T4N0M0	Positive	Positive	1.6	25.4
3	Breast (ductal)	T4N2M1	Negative	Negative		
4	SCCHN	T3N2M0	Positive	Positive	3.5	46.8
5	Breast (ductal)	T4N1M0	Positive	Positive	0.3	2.7
6	Breast (ductal)	T4N2M1	Positive	Positive	1.5	3.9
7	Breast (ductal)	T3N2M0	Positive	Positive	1.7	9.5
8	SCCHN	T4N0M0	Positive	Positive	0.7	24.2
9	SCCHN	T3N1M0	Positive	Positive	1.0	32.0
10	SCC cervix	FIGO IIIB	Negative	Positive	NA	NA
11	Breast (ductal)	T4N2M0	Positive	Positive	1.4	5.2
12	SCC bronchus	T2N0M1	Negative	Negative		
13	SCCHN	T3N2M0	Positive	Positive	0.6	9.0
14	SCCHN	T3N0M0	Positive	Positive	1.6	53.0
15	SCC bronchus	T3N0M1	Positive	Positive	2.6	16.7
16	Glioma (AA)	Inoperable	Negative	Positive	NA	NA
17	Glioma (GBM)	Inoperable	Negative	Positive	NA	NA

^a^Tumor uptake determined from ROI on 72-h whole-body scan

^b^Percentage injected dose/kg calculated from estimated tumor volume

*SCC*, squamous cell cancer; *AA*, anaplastic astrocytoma (grade III); *GBM*, glioblastoma multiforme (grade IV); *NA*, not assessable (tumor uptake was only measurable from whole-body scans)

The second lesson was a remarkably large variability of liposome uptake observed in the tumors, even after accounting for tumor size. In contrast, uptake in the main organs (liver, spleen, lungs, kidneys) was rather uniform between different patients. Although no data were available to explain this heterogeneity, variability in tumor vascularization and local inflammation were proposed as plausible explanations, based on preclinical and other clinical studies. The use of radiolabeled liposomes to predict drug uptake in patients, in other terms patient stratification, was proposed by the authors as a way to improve response rates in phase II clinical studies.

**Fig. 2.**
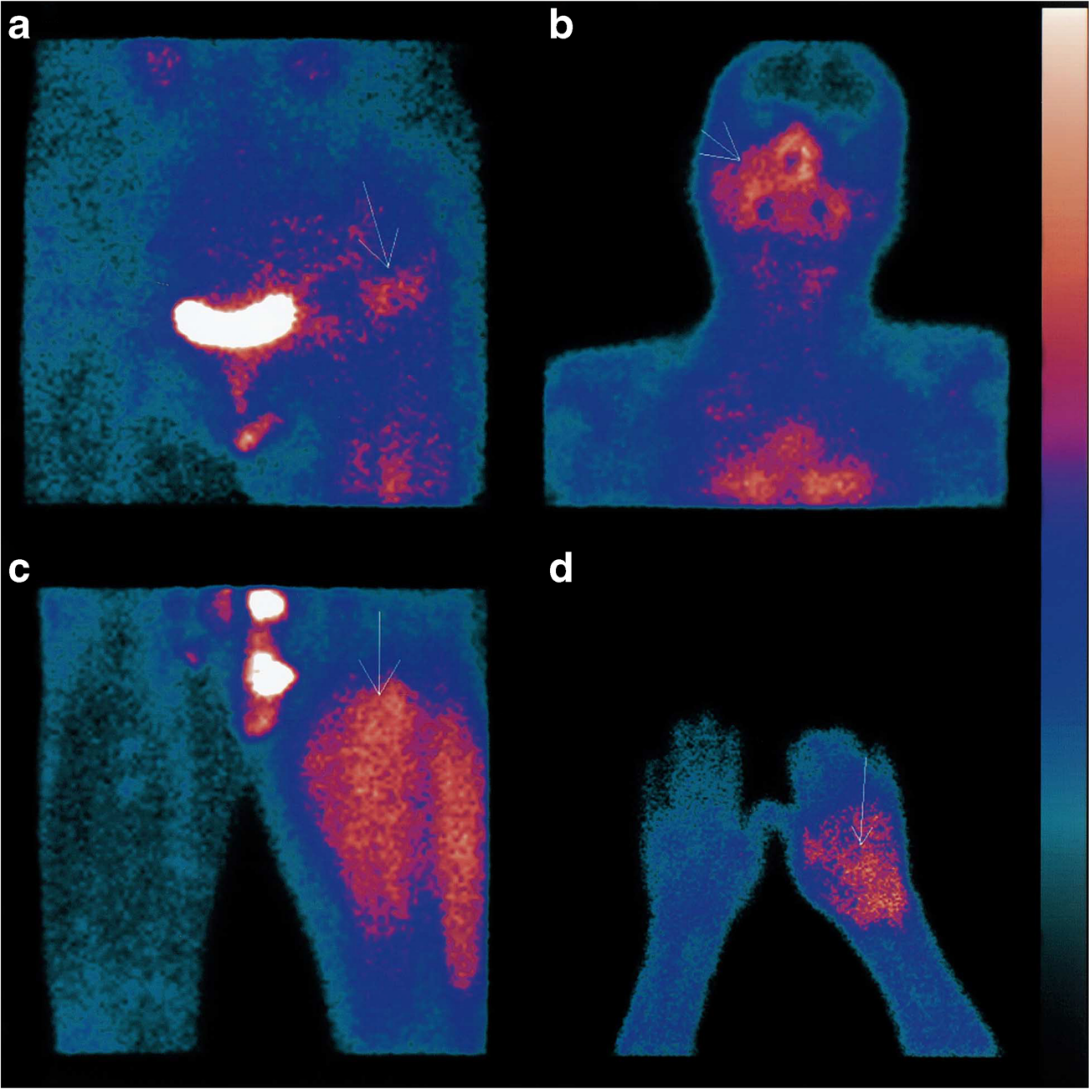
Scintigraphic planar images from four patients with sarcoma administered ^99m^Tc-labeled liposomal doxorubicin (Caelyx®). **a** Clockwise from top left: fibrosarcoma of the iliac region, **b** angiosarcoma of the maxillary andrum, **c** Ewing sarcoma of the femur, **d** Kaposi sarcoma of the palmar area. Adapted with permission from Koukourakis *et al.* [21].

A common feature of the studies described above is that the liposomes contained no other cargo than the radiotracer. The first clinical studies of radiolabeled liposomes containing a therapeutic drug were published in 1999 and 2000 by Koukourakis *et al.* [20, 21]. These pilot studies aimed at evaluating the combination of liposomal doxorubicin (Caelyx®) with radiotherapy. In the first study, nine patients with non-small cell lung cancer (NSCLC) and seven patients with HNC were administered Caelyx® radiolabeled with Tc-99m and imaged by planar gamma scintigraphy. Accumulation of the liposomes in the tumor sites was observed 2 h after administration, with tumor-to-blood ratio increasing after 10 h. In the NSCLC patients, liposomal uptake in the tumors correlated with the degree of tumor micro-vascularization, showing the presence of the EPR effect. The second study describes the same experiment in seven patients presenting locally advanced sarcomas [21] (Fig. 2). The apparent absence of liposomal drug-related toxicity and the high response rate (4 complete responses out of 7 patients) were considered encouraging, despite the low number of subjects and the absence of a control group. Although no measurement of doxorubicin levels by either imaging or biopsy was described, scintigraphy showed accumulation of Tc-99m at tumor sites previously determined by CT or bone scans, on average 2.8-fold more than in neighboring normal tissue. This again demonstrates the increased uptake of liposomes in tumors in humans. Finally, another study described liposomal mifamurtide (Mepact®) radiolabeled with Tc-99m and administered to four cancer patients for pharmacokinetic analysis, showing accumulation in lung metastases in two patients [22].

More recently, Giovinazzo *et al.* [23] investigated an indirect approach to imaging liposomal drug delivery, using Tc-99m sulfur colloid (TSC, Technecoll®) to inform on the distribution of PEGylated liposomal doxorubicin (Doxil®). TSC and other Tc-99m-labeled colloids are clinically approved and routinely used for lymphoscintigraphy and staging of cancers [42]. Since the uptake of PEGylated liposomes is at least in part mediated by the mononuclear phagocyte system (MPS), the hypothesis was that the biodistribution of TSC, which is also cleared through the MPS, would mirror that of Doxil®. Thus, TSC uptake might be used to predict the uptake of Doxil® and thereby inform on the potential efficacy and/or toxicity of this drug, in particular the risk of developing palmar-plantar erythrodysesthesia (PPE). Ten patients with epithelial ovarian cancer were administered TSC, 1 week before commencing treatment with liposomal doxorubicin. Imaging of the spleen, liver, and hands was done by scintigraphy and SPECT/CT. Levels of TSC in the hands were highly variable from one patient to another and did not correlate with blood TSC levels, suggesting variability in MPS activity. After finding a positive correlation between blood levels of TSC and encapsulated doxorubicin, the authors derived a formula to estimate doxorubicin levels in the hands based on TSC measures only. This estimated value positively correlated with clinical grades of PPE severity. The main advantage of this indirect approach is that it is based on a clinically approved product and could potentially be used as a general predictor of uptake for nanomedicines that are cleared through the MPS. On the other hand, it would still require initial correlation studies to be undertaken for each nanomedicine, whereas radiolabeling of the therapeutic nanomedicine directly and specifically informs on the uptake of the drug.

### Infection and Inflammation

In an interesting departure from cancer studies, Dams *et al.* [24] evaluated the use of ^99m^Tc-labeled PEGylated liposomes for the detection of infection and inflammation, as an alternative to the clinical standards represented by radiolabeled immunoglobulin G (IgG) and white blood cells. Here, each patient acted as their own control, being first administered the liposomes and 24 h later ^111^In-labeled IgG. Several interesting findings arose from this study. From a diagnosis perspective, there was excellent concordance between the results from the scans with Tc-99m-labeled liposomes and In-111-labeled IgG, with discordance in only 1 out of 35 patients. The calculated specificity was identical for both methods, and the sensitivity was higher with the liposomes. The liposomes also allowed better delineation of the foci of accumulation than the IgG in some patients, presumably because of the lower rate of reverse diffusion of the liposomes into the blood pool. It should be noted that the suspected foci were predominantly musculoskeletal and the radiolabeled liposomes failed to detect the single case of endocarditis in the study, highlighting a limitation of this approach. As expected, the PEGylated liposomes showed a long circulation time, with signal in the blood pool still visible 24 h after administration. Although the concordance study required the use of a shorter-lived isotope for the first scan to allow a second scan as quickly as possible, one might consider the use of a longer-lived isotope in future studies. This would allow scanning the patient later, potentially improving the signal-to-background ratio and thereby facilitating diagnosis. Later scans may also allow monitoring the response to treatment. Alternatively, modifications of the physico-chemical properties of the liposomes might allow faster clearance from the circulation for more rapid increases in target-to-background ratios, resulting in earlier diagnosis. The authors considered radiolabeled liposomes as a diagnostics tool for detecting infectious or inflammatory foci. In view of the good performance of the Tc-99m-labeled PEG liposomes in detecting such foci, it is clear that radiolabeled liposomes could also be used as theranostic agents by loading them with antibiotics or anti-inflammatory drugs. This is particularly true for antibiotics because sub-optimal drug concentrations at the site of infection can lead to the appearance of bacterial resistance. Radiolabeled liposomes could help estimate the amount of antibiotic reaching the infectious foci and allow rapid adjustments of the therapeutic schedule.

### Respiratory Diseases

An example of the aforementioned approach is given in a study by Weers *et al.* [25], using a Tc-99m-labeled liposomal formulation of the aminoglycoside amikacin, administered through a nebulizer. In this case, using aerosolized liposomes was intended to provide a slow-release formulation, reducing dosing frequency, to increase drug penetration through the bacterial biofilm and to reduce systemic exposure to the drug. The study was performed in three healthy subjects and showed that nearly 40 % of the deposited liposome dose was still present in the lungs 48 h after administration, indicating longer retention compared to non-encapsulated drugs. It also clearly showed the mucociliary escalator in action, with a large fraction of the deposited dose ending up in the stomach after being cleared upwards from the airways and swallowed. Although this radiolabeling approach could potentially have been useful for larger clinical trials, development of this liposomal antibiotic (Arikace™) appears to have halted in phase III. An earlier study of radiolabeled nebulized liposomes had also shown longer retention of the liposome-encapsulated tracer [26]. Bhavna *et al.* [27] compared the distribution of two Tc-99m-labeled, inhaled formulations of salbutamol (a long-acting β2 agonist used in asthma and chronic obstructive pulmonary disease management) with different particle sizes. The hypothesis was that a reduced particle size would lead to improved drug delivery by increasing deposition in the peripheral lung alveoli and reducing uptake by alveolar macrophages. Although the latter aspect was not described in the study, scintigraphic images reveal deeper penetration of the smaller-sized formulation (average particle size 60 nm) compared to the commercial formulation with a particle size of around 10 μm, as expected for inhaled particles [43]. Despite intense research efforts in nanomedicines for drug delivery to the lungs [44], very few clinical trials appear to have made use of the possibilities offered by non-invasive imaging techniques.

## Clinical Studies Using PET Imaging

The most commonly used positron-emitting radionuclides in clinical studies are fluorine-18 (F-18, *t*_1/2_ = 110 min), rubidium-82 (Rb-82, *t*_1/2_ = 1.3 min), carbon-11 (C-11, *t*_1/2_ = 20.3 min), gallium-68 (Ga-68, *t*_1/2_ = 67.8 min), copper-64 (Cu-64, *t*_1/2_ = 12.7 h), and zirconium-89 (Zr-89, *t*_1/2_ = 3.27 day). Positrons emitted from PET radioisotopes travel up to a short distance (positron range, up to a few mm) before encountering an electron, at which point an annihilation event produces two 511-keV gamma rays at a near 180° angle. This pair of coincident gamma photons is detected by the PET camera, in which detectors are arranged in several static rings, providing 3D images. The coincidence detection allows to dispense with physical collimators used in SPECT/planar scintigraphy, thereby increasing the sensitivity of PET scanners by several orders of magnitude over SPECT scanners, in the range of 10^−11^ to 10^−12^ mol/l of radiotracer, as well as the temporal resolution [35]. The lack of collimators also results in signals that are more easily quantifiable than those from gamma-emitting isotopes, and a maximum spatial resolution of approximately 5 mm. Despite these advantages, only two recent clinical studies of nanomedicines using PET have had results published as of April 2018, both in the field of oncology.

In the context of a clinical trial (NCT01304797) of MM-302, a formulation of PEGylated liposomal doxorubicin targeted against human epidermal growth factor receptor 2 (HER2), Lee *et al.* [28] selected 19 patients with metastatic breast cancer for an imaging study using MM-302 radiolabeled with Cu-64. Current clinically approved formulations of PEGylated liposomal doxorubicin (Doxil®/Caelyx®, Myocet®) do not possess targeting moieties and rely solely in the EPR effect. MM-302 is targeted towards HER-2 with the objective of increasing delivery of doxorubicin in HER2-overexpressing cells rather than in macrophages, as observed in a preclinical study [45].

**Fig. 3.**
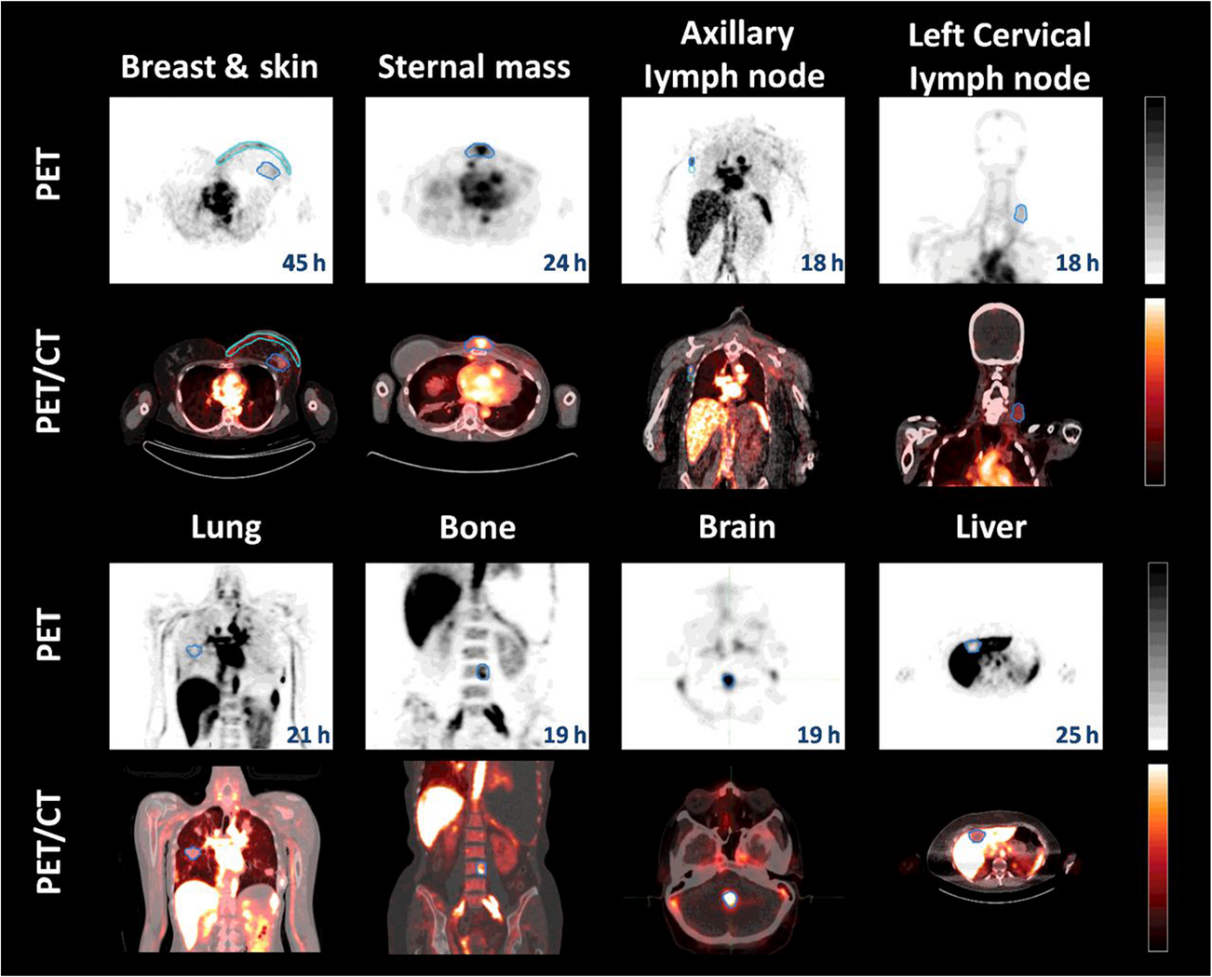
Representative PET and fused PET/CT images of [^64^Cu]-MM-302 in lesions at different anatomic locations. The regions of interest used to measure tumor deposition of [^64^Cu]-MM-302 are shown in blue or turquoise outlines. Adapted with permission from the American Association for Cancer Research: Lee *et al.* [28].

The authors sought to determine whether the amount of drug reaching the metastases would correlate with therapeutic efficacy. Treatment with MM-302 was given along with trastuzumab (a clinically approved anti-HER2 monoclonal antibody). The chelating and loading agent 4-DEAP-ATSC [46] was used to radiolabel MM-302 with Cu-64, with a target dose of 400 MBq per patient. The maximum Cu-64 activity remaining in the patients would be 108 MBq after 24 h and 29 MBq after 48 h, potentially sufficient for PET imaging at this time point. Results from PET imaging (Fig. 3) showed that [^64^Cu]MM-302 remained in the circulation for over 24 h, and thereafter accumulated mostly in the liver and spleen, in good agreement with preclinical data.

The authors state that free Cu-64 was not detectable in the patients selected for more detailed analysis. This is not surprising, because free Cu-64 has high affinity for liver and spleen tissues and rapidly accumulates in these organs [47]. Therefore, assessing the stability of the Cu-64 radiolabeling of MM-302 based on PET images is not straightforward. Based on preclinical data showing that the biodistribution of MM-302 could be affected by treatment with cyclophosphamide, the patients selected for the imaging study were taken both from a group receiving MM-302 and trastuzumab and from a group receiving cyclophosphamide in addition. However, no differences in drug uptake were observed between these two groups, leading the patients to be analyzed as a single group for the rest of the study. There were several important results from the study. The first is the large heterogeneity in drug uptake not only between subjects but also between lesions within subjects. This high variability of the EPR effect is particularly important from a therapeutic point of view, since metastases exposed to insufficient drug concentrations could serve as starting points for further dissemination of cancer cells, potentially negating the initial benefits of the treatment. This led the authors to stratify the patients according to the amount of nanomedicine present in the lesion with the lowest uptake. Although the study was underpowered to show a correlation between MM-302 uptake and progression-free survival, an encouraging trend was observed that would warrant the enrollment of additional patients. A second lesson was the good agreement between clinical and preclinical data, both in overall nanomedicine distribution and in drug concentrations in the tumors. This should strengthen the case for clinical trials when imaging-based preclinical data are encouraging. Furthermore, the authors found no correlation between drug concentrations at the tumors and either drug concentrations in the blood or tumor size. This means that blood sampling will not be predictive of efficacy and highlights the added value of quantitative whole-body imaging techniques. A limitation of this study may paradoxically reside in the use of Cu-64 as an imaging agent. PET imaging has also been used by several groups [48–50], to show that the maximal tumor uptake of PEGylated liposomes in preclinical models occurs within 24–48 h post-administration, but imaging at later time points (*e.g.*, 72 h) improves signal-to-background ratio. In practice, the half-life of Cu-64 limits the imaging window to approximately 48 h post-administration and even this duration requires high starting amounts of radioactivity. The use of longer-lived PET isotopes, such as Zr-89 (*t*_1/2_ = 3.27 day) or Mn-52 (*t*_1/2_ = 5.59 day), should overcome this barrier.

The other clinical study of a PET-radiolabeled nanomedicine was conducted by Phillips *et al.* [29], using fluorescent nanoparticles conjugated to iodine-124 (I-124, *t*_1/2_ = 4.18 day), for optical and PET imaging. The nanoparticles were also conjugated to an integrin-targeting peptide and engineered to promote renal clearance, and were therefore expected to have a distinct biodistribution pattern compared to liposomal nanomedicines. Indeed, the authors observed rapid clearance of the nanoparticles with most of the activity eliminated within 72 h and no accumulation in the liver and spleen. This is an interesting feature compared to liposomal nanomedicines, where liver and spleen accumulation complicates the visualization of nearby tumors. Although the study focused mainly on the safety and stability of the nanoparticles, accumulation of the nanoparticles was observed at tumor sites in some patients, with increasing target-to-background ratios over time but already observable within 4 h of administration. This favorable pharmacokinetic profile may enable faster clinical decisions. On the other hand, the nanoparticles were observed to accumulate in the renal cortices of a patient known to have chemotherapy-induced kidney inflammation, potentially complicating the differentiation between tumor areas and inflammatory lesions. There would be no reason to expect off-tumor inflammation in preclinical models of cancer, and therefore such a chance observation could only be made in a clinical study. This shows the value of incorporating whole-body imaging at the earliest opportunity in clinical studies of nanomedicines. Furthermore, the combination with an optical probe is an excellent choice for clinical applications of multimodal imaging, allowing tumor localization by PET to be followed by fluorescence-guided surgery for improved tumor resection, ultimately resulting in improved patient outcomes.

## Clinical Studies Using Magnetic Resonance Imaging

In magnetic resonance imaging, the subject is placed in a powerful magnetic field, which will align magnetically active nuclei (most commonly hydrogen from water molecules) either parallel or anti-parallel to the magnetic field. The small difference in the number of nuclei aligning in each direction gives rise to the magnetic resonance imaging (MRI) signal. A pulsed radiofrequency can then be used to temporarily disturb the alignments of the nuclei, and the relaxation time back to the original position is measured. This relaxation time is dependent on the environment of the nuclei, for example, hydrogen nuclei in a fat-rich environment, or with short distance of an iron oxide nanoparticle, have shorter relaxation times than those in an aqueous environment. It is important to note that when using contrast agents in MRI, the measured signal arises not directly from the imaging agent but from the changes in magnetic properties the agent induces in its local environment. MRI has a much lower sensitivity than nuclear imaging techniques and requires imaging agent concentrations in the range of 10^−3^ to 10^−5^ mol/l, which can have pharmacological effects. Although MRI avoids the use of ionizing radiation, the inherent presence of MR-active nuclei means that at least two imaging sessions are necessary when imaging drug delivery: one before the administration of the MR-detectable agent, and one at a suitable time after administration. In addition, accurate quantification of the signal derived from contrast agents in MRI, particularly those based on superparamagnetic iron oxide materials, is difficult. On the other hand, the spatial resolution of clinical MRI is approximately 1 mm, providing far more detailed images than PET or SPECT [35].

**Fig. 4.**
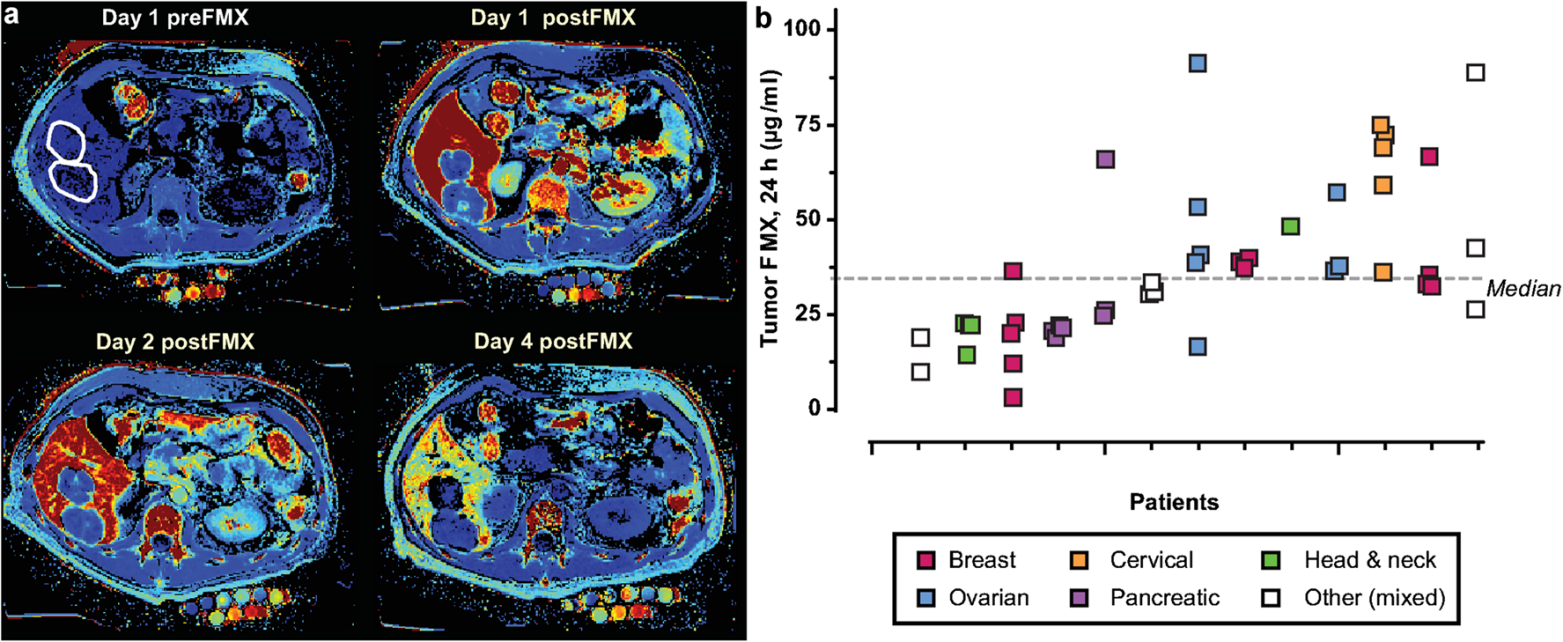
**a** Representative pseudocolored relaxometric R2* maps derived from patient images before and after administration of iron oxide nanoparticle (Ferumoxytol®, FMX). Approximate lesion locations are indicated by white lines in the image before FMX dosing. **b** Extrapolated FMX concentrations in individual patients 24 h after administration. Adapted with permission from the American Association for Cancer Research: Ramanathan *et al.* [30].

In a recent study, Ramanathan *et al.* [30] described an interesting approach to image-guided drug delivery. The aim was to use the tumor deposition of Ferumoxytol, a carboxy-dextran-coated superparamagnetic iron oxide nanoparticle with long circulation time and affinity for macrophages [51], as a surrogate marker for tumor deposition of nal-IRI (MM-398, Onivyde®), a nanoliposomal formulation of the topoisomerase I inhibitor irinotecan. Both Ferumoxytol and nal-IRI are clinically approved products. The driving idea was that the amount of nanomedicine reaching the tumor would depend more on the vascular permeability at the tumor site and the average particle size than on the specific composition of the nanomedicine, so that iron nanoparticles and liposomes with otherwise comparable pharmacokinetics would reach the tumor in similar amounts. This study was performed in 13 patients with solid tumors, of which 9 were also assessed for treatment response by CT. Ferumoxytol uptake was quantified by T2* imaging (Fig. 4), and irinotecan levels were measured from patient biopsies. There was no statistically significant correlation between Ferumoxytol uptake and irinotecan levels.

In a similar two-class approach to that used by Lee *et al.* [28] in the PET study of MM-302 described above, the authors classified lesions according to their Ferumoxytol uptake (below or above the median of uptake in all lesions). Using this approach, lesions with above-median Ferumoxytol uptake showed significantly improved changes in size after nal-IRI treatment over those with below-median uptake. Another major finding of this study was the high variability of Ferumoxytol between patients and between lesions in a given patient, echoing the high variability of the EPR effect reported for Tc-99m- and Cu-64-labeled PEGylated liposomes [19, 28]. This MRI-based study takes a “companion diagnostic” approach akin to that used in the TSC/Doxil® combination study by Giovinazzo *et al.* [23]. Since the uptake of one drug is used as an indirect reflection of the uptake of a second therapeutic drug, there is an inherent possibility of pharmacokinetic/biodistribution differences between the two drugs. Correlations must therefore be established in preliminary studies for each envisaged reporter/drug combination before the pair can be used in the clinic. However, the use of clinically approved imaging agents could easily be integrated into existing treatment protocols by not having to go through the same regulatory hurdles as theranostic agents, which could be considered as new drugs and therefore require full regulatory approval.

Two other clinical trials (NCT02022644 and NCT03086616) of nal-IRI that include image-guided drug delivery are planned. Convection-enhanced delivery (CED) of nal-IRI to the brain of pediatric and adult patients with glioma will be followed in real time by MRI, using co-administered gadolinium-based contrast agents.

## Clinical Studies Using Ultrasound Imaging

Ultrasound imaging relies on sending sound waves through the body and recording the reflected waves to produce a high-resolution 2D image. This is done with a hand-held probe, making it a widely available and very cost-effective technique, but is mostly limited to soft tissue imaging [35]. There is a large body of preclinical work on focused ultrasound (FUS)-mediated drug delivery, especially using thermosensitive liposomes, with the imaging aspect typically performed by ultrasound or MRI. For further information on this topic, the reader is directed to recent and extensive reviews [52–54]. We have found only one report related to ultrasound-guided drug delivery in humans. Lyon *et al.* [31] recently described the protocol of an ongoing (as of April 2018) clinical trial of ThermoDox®, a thermosensitive liposomal formulation of doxorubicin with FDA approval for investigational use. By using low-intensity FUS to visualize liver tumors and induce mild hyperthermia, the aim is to selectively increase the concentration of doxorubicin at the tumor sites. Doxorubicin levels will be measured by high-performance liquid chromatography in biopsied tumor tissues before and after application of FUS. Only the first few patients are to undergo tumor biopsy before FUS, to establish an average tumor concentration of doxorubicin prior to FUS application. The authors describe this as a way of reducing the number of invasive procedures performed on the patients. However, considering the variability of liposomal uptake in tumors highlighted in the MM-302 study [28], we caution that this approach might yield misleading results. It would be preferable, especially for a pilot study, to determine pre-FUS doxorubicin concentration in as many patients as possible to adequately quantify the effect of FUS on liposomal drug release. Other imaging modalities such as [^18^F]FDG PET-CT and dynamic-contrast enhanced (DCE)-MRI are included in the study, but aimed at establishing baseline tumor imaging and potential response to treatment rather than evaluating drug delivery. Results from this study have yet to be published; however, it will be of considerable interest to see whether a non-invasive and non-ionizing method such as FUS can be used to increase doxorubicin concentrations above therapeutic threshold specifically in cancerous tissue to minimize the effect on healthy tissues.

## Conclusions and Perspectives

Imaging has been successfully used in humans to study the biodistribution and pharmacokinetics of nanomedicine-based drug delivery systems, demonstrating its value for this purpose. Significant findings from these studies include solid proof that (1) EPR as the most common uptake mechanism for nanomedicines in tumors/inflamed tissues is a real phenomenon in patients and (2) the EPR effect is highly heterogeneous, between diseases, patients, and even lesions within a single patient. This heterogeneity may underlie the fact that nanomedicines have not always shown superiority in clinical therapeutic activity over conventional drugs, despite an improved safety profile [11]. Indeed, an absence of therapeutic effect does not imply absence of nanomedicine accumulation in tumors. This is particularly relevant for metastatic cancers, as nanomedicine accumulation only at certain tumor sites may not be sufficient for an overall survival benefit to the patient. Moreover, recent clinical studies have demonstrated a clear correlation between nanomedicine accumulation levels in target tissues (*e.g.*, tumors) and therapeutic effect, supporting the use of imaging to identify patients that will respond to the treatment. An important concept that arises from these studies is the development of companion diagnostics, based on clinically approved imaging agents that correlate with nanomedicine biodistribution. Another conclusion from this review is that most of the studies to date have been in the oncology field, leaving further opportunities for image-guided drug delivery studies beyond this area, particularly in the fields of infection and respiratory medicine.

A general limitation of the nanomedicine-imaging approaches described in this review is that they are in practice indirect methods. Indeed, the imaging agent is most frequently attached to the carrier rather than to the drug, whereas clinically the most relevant information is the localization of the drug itself, which may differ from that of the carrier after release. This would generally require chemical modification of the drug. Furthermore, it is extremely challenging to differentiate between nanocarrier-bound and released drug by non-invasive imaging. This may be possible by optical imaging, especially for intrinsically fluorescent drugs, *in vitro* and possibly at the preclinical level, but not in humans [55]. An option is co-loading liposomes with a drug and a magnetic resonance (MR) contrast agent, as the relaxivity of the encapsulated and non-encapsulated MR contrast agent will differ [56], but this method suffers from the inherent limitations of MRI discussed above (sensitivity, challenging whole-body detection/quantification). Furthermore, it will not inform on drug distribution after release and at later times. Nuclear imaging modalities may be helpful in this context, for example, by loading radiolabeled drugs into nanocarriers; however, the half-lives of the radioisotopes most amenable to radiolabeling of small molecules (*e.g.*, C-11 and F-18) are too short to match the biological half-lives of nanomedicines. In this context, a recent preclinical study demonstrated the concept of radiolabeling both the liposome carrier and the encapsulated drug with PET radionuclides [57]. Another possibility is to use multi-isotope SPECT and image both the carrier and the drug orthogonally; however, this is only possible when using radionuclides with different gamma emission energies (*e.g.*, Tc-99m and In-111). Longer imaging windows are attainable by using metal-chelating drugs and radiometals such as Zr-89 [48]; however, this raises another issue, which is the stability of the free radionuclide/label. Even the longer-lived and covalently bound radioiodine (I-124, I-125, I-131) labels are susceptible to deiodination *in vivo*. As mentioned previously, the accumulation of the free radiolabel in specific anatomical locations (*e.g.*, the thyroid for radioiodine, bones for Zr-89, or pancreas for Mn-52 [58]) may inform on the extent of drug release from the carrier. Even this requires the assumption that the rate of release of the radiolabel from the drug is similar to the drug release from the carrier and will not inform about drug localization.

Despite all these issues, it is clear that among the different imaging techniques available, nuclear medicine techniques have been the most used to date, most likely due to their whole-body capabilities and excellent quantification properties. Importantly, their high sensitivity also allows imaging with sub-therapeutic nanomedicine doses in the microdose range (1 % of the therapeutic dose), which facilitates clinical translation. MRI and US circumvent the use of radioactivity and provide higher spatio-temporal resolution, at the expense of lower sensitivity and higher imaging agent doses. In addition, performing quantitative whole-body imaging with MRI and US is a more complex process than with nuclear imaging. For these reasons, it is likely that future clinical studies in this area will include nuclear imaging as the main quantitative method to assess nanomedicine/drug concentration *in vivo*. The recent study by Lee *et al.* [28] using [^64^Cu]MM-302 is an excellent example of the way clinical image-guided drug delivery studies should be conducted nowadays, particularly for its approach to patient stratification. We believe that incorporating imaging into studies of nanomedicine drug delivery will undoubtedly provide valuable additional information that standard techniques such as blood sampling and biopsy cannot provide. This will greatly benefit the clinical development of new nanomedicines and help achieve the full potential of those already developed.
